# Complete Correction following Temporary Hemiepiphysiodesis using 2-hole Reconstruction Plates among Skeletally Immature Patients with Angular Deformities around Knees: A Descriptive Cross-sectional Study

**DOI:** 10.31729/jnma.8494

**Published:** 2024-03-31

**Authors:** Tarun Kumar Rajbhandary, Subhash Regmi, Yam Prakash Gurung, Bibek Banskota, Satish Prasad Barnawal, Ashok Kumar Banskota

**Affiliations:** 1 Department of Orthopaedics, Hospital and Rehabilitation Center for Disabled Children (HRDC), Banepa, Kavre, Nepal; 2 Department of Orthopaedics, B&B Hospital Pvt. Ltd., Gwarko, Lalitpur, Nepal

**Keywords:** *bone plates*, *genu valgum*, *genu varum*

## Abstract

**Introduction:**

Studies have shown that 2-hole reconstruction plates can be used effectively as tension band plates for temporary hemiepiphysiodesis. However, limited data is available regarding the effectiveness of such plates in terms of complete correction rates. This study was aimed to find out the prevalence of complete correction following temporary hemiepiphysiodesis using 2-hole reconstruction plates among skeletally immature patients with angular deformities around knees.

**Methods:**

A descriptive cross-sectional study was carried out among skeletally immature patients with angular deformities around knees undergoing temporary hemiepiphysiodesis after getting approval from the Institutional Review Committee (Reference number: B&BIRC-23-05). The data were collected between 1 January 2012 to 31 December 2018. All skeletally immature patients with angular deformities around knees undergoing temporary hemiepiphysiodesis using 2-hole reconstruction plates were included. Patients who required additional procedures or implants for deformity correction and those who did not provide consent were excluded. Convenience sampling method was used. Point estimate at 95% Confidence Interval was calculated.

**Results:**

Among 102 patients, 66 (64.70%) patients achieved complete correction (59.97-69.43 at 95% Confidence Interval). Mean age of the patients was 8.21±3.11 years and 43 (65.15%) were males and 23 (34.84%) were females.

**Conclusions:**

The prevalence of complete correction following temporary hemiepiphysiodesis using 2-hole reconstruction plate among skeletally immature patients with angular deformities around knees was lower than that reported in other international studies.

## INTRODUCTION

Angular deformities around knees in children can either be physiological or pathological; physiological deformities improve overtime, but pathological deformities require treatment.^1^ Several treatment options have been described, such as temporary hemiepiphysiodesis, timed permanent hemiepiphysiodesis, osteotomies, and Ilizarov ring-fixators.^2-4^ Out of which, temporary hemiepiphysiodesis, using staples, percutaneous screws, or tension band plates, has gained popularity because of less invasiveness.^5,6^ Studies have also found that temporary hemiepiphysiodesis using figure-of-eight plates superior to staples or screws.^5,7^ However, figure-of-eight plates are costly and may not be available in low-income countries.^6,8^

Studies have shown that 2-hole reconstruction plates can be used effectively as tension band plates, an alternative to figure-of-eight plates, for temporary hemiepiphysiodesis.^7,9^ However, limited data is available regarding the effectiveness of such plates in terms of complete correction rates.

This study was aimed to find out the prevalence of complete correction following temporary hemiepiphysiodesis using 2-hole reconstruction plates among skeletally immature patients with angular deformities around knees.

## METHODS

A descriptive cross-sectional study was carried out among skeletally immature patients with angular deformities around knees undergoing temporary hemiepiphysiodesis after getting approval from the Institutional Review Committee (Reference number: B&BIRC-23-05). The data were collected between 1 January 2012 to 31 December 2018. All skeletally immature patients with angular deformities around knees undergoing temporary hemiepiphysiodesis using 2-hole reconstruction plates were included. Patients who required additional procedures or implants for deformity correction and those who did not provide consent for the inclusion in the study were excluded. Convenience sampling method was used. The sample size was calculated using following formula:


$$\begin{array}{ll} \text{n} & ={\text{Z}}^{2}\times \frac{\text{p}\times \text{q}}{{\text{e}}^{\text{2}}}\\ & =1.96^{2}\times \frac{0.86\times 0.14}{0.07^{2}}\\ & =95 \end{array}$$

Where,

n = minimum required sample size
Z = 1.96 at 95% Confidence Interval (CI)
p = prevalence of complete correction taken as 86% from the previous study^9^
q = 1-p
e = margin of error, 7%

The calculated minimum required sample size was 95. This study included a total of 102 patients. All patients underwent measurement of angular deformities (in degrees) using plain radiographs: a full-length standing anterior-posterior (AP) of both legs, with the patella facing forward.^9^ The measurements were done by two fellowship trained surgeons in pediatric orthopedics and the disagreements were resolved by senior surgeon. Patients with metabolic issues were optimized prior to surgery. Surgeries were performed by the same group of surgeons with more than three years of experience in pediatric orthopedics following standard technique described by Stevens.^3^ All patients underwent ambulatory exercises immediately after operation as tolerated under the guidance of trained physiotherapists. All patients were followed every four months. The plate and screws were removed after complete correction was achieved.

Primary outcome measure was prevalence of complete correction, which is defined as neutralization of the mechanical axis.^9^ Secondary outcome measures were mean duration to achieve complete correction (in months) and prevalence of complications, which included screw breakage, ligament laxity, rebound deformity, and revision surgeries. Following data were recorded in an electronic proforma; the age, gender, geographical distributions, etiology, involved knee, varus or valgus deformities, correction status, correction time, and complications.

The data were analyzed using Microsoft Excel version 2019. As the sample size was more than 30, the data wasassumedtobe normally distributed.Continuous data were reported as mean±standard deviation (SD) and categorical data were reported as number (percentage). Point estimates at 95% CI were calculated.

## RESULTS

Among 102 patients, 66 (64.70%) (59.97-69.43 at 95% CI) patients achieved complete correction. Mean age of the patients was 8.21±3.11 years and 43 (65.15%) were males and 23 (34.84%) were females. The baseline characteristics of patients who achieved complete correction was calculated (Table 1).

**Table 1:** Baseline characteristics of patients who achieved complete correction (n = 66).

Characteristics	n (%)
**Geographical distribution**	
Mountain	6 (9.09)
Hill	26 (39.39)
Terai	30 (45.45)
Others	4 (6.06)
**Etiology**	
Metabolic	31 (46.96)
Congenital	29 (43.93)
Trauma	4 (6.06)
Infection	2 (3.03)
**Involved knee**	
Bilateral	57 (86.36)
Right	5 (7.57)
Left	4 (6.06)
**Deformity**	
Varus	36 (54.54)
Valgus	30 (45.45)

The mean follow-up duration was 31.96±9.6 months. The mean time to achieve complete correction was 19.25±8.39 months. The overall prevalence of complications was 23 (34.84%), including 6 (9.09%) screw breakage, 10 (15.15%) revision surgeries, 5 (7.57%) rebound deformity and 2 (3.03%) ligamentous laxity.

## DISCUSSION

This study identified that the prevalence of complete correction following temporary hemiepiphysiodesis using 2-hole reconstruction plate among children with angular deformities around knees was 66 (64.70%). The finding was lower than that reported in international studies, which was around 86-95%.^7,9^ A study conducted in Turkey including 77 patients with 166 knees observed complete correction in around 95% of the cases.^7^ Similarly, other study conducted in United States including 21 patients with bilateral angular deformities around knees observed complete correction in around 86% of the cases.^9^ The higher prevalence of complete correction observed in those studies could be due to regular follow-up of the patients, as they have not observed any patients with overcorrection. In our study, the lower prevalence of complete correction was due to the tendencies of patients following up late and presenting with overcorrection.

This study also identified that the mean time to achieve complete correction was 19.25±8.39 months. The finding was slightly higher than that reported in other international studies, which was around 11-18 months.^3,7^ A study conducted in the United States including 34 patients with 64 deformities around knees observed complete correction in an average of 11 months. Similarly, other study conducted in Turkey observed complete correction at an average of 18±8 months.^7^ These findings could be due to patients' compliance to follow-up visits and early recognition and recording of complete correction. Furthermore, the finding was also higher that that reported in the study conducted in similar settings, which was around 15.6 months.^5^ A study conducted in India including 24 patients observed complete correction at an average of 15.6 months.^5^ However, they have included patients with mean age of 5.25 years compared to 8.21 years in our study.^5^ This suggests that inclusion of younger patients might result in early correction of the deformity.

This study also identified that the overall prevalence of complications among those who achieved complete correction was 23 (34.84%). The finding was similar to that reported in other international studies, which ranges from 6-41%.^3,7,9^ Although there were considerable variations among studies regarding the selection of complications, common complications reported include screw breakage and rebound deformity. In this study, the prevalence of screw breakage was around 6 (9.09%). The finding was slightly higher than that reported in international studies, which was around 5-7%.^3,7,9^ In contrast, the finding was similar to that reported in study conducted in similar settings, which was around 8.3%.^5^ This suggests that the higher prevalence of screw breakage could be due to the quality of implants and poor patient compliance to rehabilitation protocols. Similarly, the prevalence of rebound deformity was around 41% in the study conducted in Turkey compared to 5 (7.57%) in this study.^7^ The reason for higher prevalence of rebound deformity in the study conducted in Turkey could be due to longer duration of follow-up which was around 10 years.^7^ This suggests that there is high risk of rebound deformity following implant removal among patients treated with temporary hemiepiphysiodesis. Furthermore, in this study, revision surgeries were required in 10 (15.15%) of the cases because of implant malposition, loosening, and prominence, and ligamentous laxity was observed in 2 (3.03%) of the cases. These complications were not reported in previous studies.^3,5,7,9^ These complications might have occurred due to the quality of implant used, surgeons' expertise and learning curve, and poor patient compliance to rehabilitation protocols.

This study had some limitations. There were risks of selection and reporting biases, as the patient selection and measurements were done by investigators involved in the study. The outcomes observed in this study cannot be generalized to all pediatric patients undergoing temporary hemi-epiphysiodesis using 2-hole reconstruction plates, as complete correction was taken as outcome of interest, and it was a single center study. A longer follow-up duration might provide insight on higher risks of rebound deformity following implant removal.^7^

## CONCLUSIONS

The prevalence of complete correction following temporary hemiepiphysiodesis using 2-hole reconstruction plateamong skeletally immature patients with angular deformities around knees was lower than that reported in other international studies.
